# Annual Meeting of the Chicago Medical Society, April 3d, 1868

**Published:** 1868-05

**Authors:** 


### ANNUAL MEETING OF THE CHICAGO MEDICAL SOCIETY, APRIL 3D, 1868.

Regular Annual Meeting of the Chicago Medical Society, Vice-President Dr. Reed in the chair. Dr. Loverin was chosen Secretary, *pro tem.*

Dr. Norman Bridge was proposed for membership. Referred to the Board of Censors.

On motion, the Society proceeded to the election of officers for the ensuing year. The Chair appointed as tellers, Drs. Ray and Macdonald.

On motion of Dr. Paoli, an informal ballot was taken for President, resulting as follows, *viz.:* whole number of votes cast 20, of which Dr. Marguerat received 9, Dr. Hatch 6, scattering 5. The regular vote was then taken, and Dr. Marguerat receiving a majority, was, on motion, declared unanimously elected President for the coming year.

The Society then proceeded to the election of Vice-President, resulting as follows: whole number of votes cast 21, of which Dr. Bogue received 16, scattering 5. On motion, Dr. Bogue was declared unanimously elected.

For Secretary and Treasurer, the number of votes cast was

21, of which Dr. Hutchinson received 9, Dr. Macdonald 12. On motion of Dr. Wickersham, Dr. Macdonald was declared unanimously elected.

The Chair appointed Drs. Davis and Wickersham to conduct the President and Vice-President to their chairs.

Dr. Marguerat thanked the Society for the honor conferred upon him, by electing him President, and would endeavor to discharge his duties to the best of his ability. Remarks were also made by Dr. Bogue to the same effect.

The newly elected President said he desired one week's consideration before naming the standing committees.

On motion of Dr. Wickersham, a vote of thanks was tendered to the retiring officers, for the very able manner in which they had discharged their duties.

On motion of Dr. Davis, the Society proceeded to the election of delegates to the American Medical Association, to be held in Washington, D.C., on the first Tuesday in May. The following named gentlemen were duly elected: Drs. Reed, Hamill, Fisher, Ross, Paoli, Holmes, and Rush.

On motion, the Society proceeded to elect delegates to the Illinois State Medical Society, to be held in Quincy, on the third Tuesday in May. The following named gentlemen were duly elected: Drs. Hildreth, Holmes, Hatch, Fitch, Bevan, Bogue, Paoli, Quales, Guerin, Hutchinson, Reed, Miller, Marguerat, and Earle.

Dr. Lyman, the retiring Secretary, presented a report, showing the expenditures for the past year ($59.46), and the amount of cash on hand ($8.93). On motion of Dr. Davis, the report was accepted and ordered to be placed on file.

On motion of Dr. Davis, the delegates to the Illinois State Medical Society were requested to invite the said Society to hold its annual meeting for 1869 in Chicago.

A motion was made and carried, that the Society postpone the discussion on diphtheria until the next meeting.

Dr. Wanzer exhibited a pathological specimen, stated by him to be an "exudation of the bowels in the form of shreds." He remarked that the patient had suffered for some years with syphilitic contamination, inclined to constipation, her bowels being neither tympanitic nor tender. Treated the patient for secondary syphilis, administering proto-iodide of mercury and iodide of potassium in proper doses. Dr. W. desired the opinion of the Society as to the character of the specimen presented.

Dr. Davis said that he was not prepared to give his opinion until the specimen had undergone a microscopic examination.

Dr. Paoli suggested that, probably, Dr. Lyman would favor the Society by a microscopic examination and report on the case at the next meeting.

Dr. Wickersham moved the following resolution, which was carried:

*Resolved*, That the delegates from this Society to the American Medical Association and to the Illinois State Medical Society be instructed to vote for no one for office at the gift of said Societies, unless the candidate is a member of a local medical society, providing there is such an organization in the county where the candidate resides.

On motion, the Society adjourned.

---

CHICAGO, *April 10, 1868.*

The Regular Meeting of the Chicago Medical Society was called to order by the President, Dr. Marguerat.

The Secretary read the proceedings of the Annual Meeting of the Society, held on the 3d inst., which proceedings were duly approved.

Dr. Davis then recommended that the Society proceed to ballot for the election of Dr. Norman Bridge as a member of the Society, which was duly carried. Number of votes cast, 8, all being for the admission of Dr. Bridge.

The President then appointed the following standing committees:

*On Ethics*—Drs. Davis, Paoli, and Ross.

*Censors*—Drs. Wickersham, Reid, and Loverin.

*Sanitary Committee*—Drs. Hatch, Trimble, and Seeley.

Under the call for pathological specimens, Dr. Bogue presented a nasal polypus, extracted from the nose of a child. Its attachment was near the posterior opening of the nostril, and had become so pendulous as to touch the tongue and somewhat obstruct the breathing. It was more firm than the ordinary soft nasal polypi.

Dr. Davis stated that he extracted a very similar polypus from a boy of 10 years, some days since, although not quite so pendulous in form as the specimen presented. This polypus being also extracted through the posterior nares.

Dr. Loverin remarked, that it had been recommended that a small portion of the inferior turbinated bone be removed along with the polypi, as it prevented their return.

Dr. Davis spoke of removing a nasal polypus from the anterior portion of the inferior turbinated bones, and of its cure by using a solution of liq. zinci chloridi gtt. iv, aqua ℥j, either injected or well snuffed up the nose. He also recommended a snuff consisting of:

℞. Pulv. Bloodroot,------------------------
" Opii,------------------------āā gr. xx
Acidi Tannici, ------------------------ ℨj.
M.

The subject for discussion, *viz.*, Diphtheria, was then taken up for consideration. The discussion was commenced by a written communication from Prof. DeLaskie Miller, read by the Secretary. The discussion that followed was participated in by Drs. Bogue, Davis, Paoli, Loverin, Hutchinson, and Marguerat; and though interesting and profitable, as a free interchange of views, it elicited no facts of importance not already before the profession.

